# Atomic-scale visualization of oxide thin-film surfaces

**DOI:** 10.1080/14686996.2018.1442616

**Published:** 2018-03-16

**Authors:** Katsuya Iwaya, Takeo Ohsawa, Ryota Shimizu, Yoshinori Okada, Taro Hitosugi

**Affiliations:** a Advanced Institute for Materials Research (AIMR), Tohoku University, Sendai, Japan; b Center for Emergent Matter Science, RIKEN, Saitama, Japan; c National Institute for Materials Research, Ibaraki, Japan; d School of Materials and Chemical Technology, Tokyo Institute of Technology, Tokyo, Japan

**Keywords:** Oxide thin films, scanning tunneling microscopy, pulsed laser deposition, 10 Engineering and Structural materials, 105 Low-Dimension (1D/2D) materials, 201 Electronics / Semiconductor / TCOs, 205 Catalyst / Photocatalyst / Photosynthesis, 212 Surface and interfaces, 306 Thin film / Coatings

## Abstract

The interfaces of complex oxide heterostructures exhibit intriguing phenomena not observed in their constituent materials. The oxide thin-film growth of such heterostructures has been successfully controlled with unit-cell precision; however, atomic-scale understandings of oxide thin-film surfaces and interfaces have remained insufficient. We examined, with atomic precision, the surface and electronic structures of oxide thin films and their growth processes using low-temperature scanning tunneling microscopy. Our results reveal that oxide thin-film surface structures are complicated in contrast to the general perception and that atomically ordered surfaces can be achieved with careful attention to the surface preparation. Such atomically ordered oxide thin-film surfaces offer great opportunities not only for investigating the microscopic origins of interfacial phenomena but also for exploring new surface phenomena and for studying the electronic states of complex oxides that are inaccessible using bulk samples.

## Graphical abstract

**Figure:**
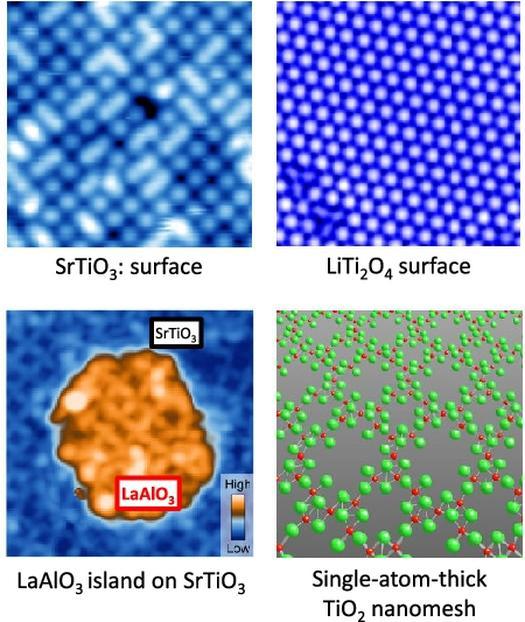

## 1. Introduction

Surfaces and interfaces are known to play crucial roles in materials science. For instance, electronic devices underpinning modern technologies greatly rely on two-dimensional (2D) electron gases formed at the interface of semiconductor heterostructures [1]. A wide range of chemical reactions in catalysts, fuel cells, and batteries occur at the surfaces and interfaces of their constituent materials [2]. Furthermore, because of the broken inversion symmetry at surfaces and interfaces, interesting physical phenomena are widely observed such as spontaneous spin polarization in strong spin–orbit coupling systems [3]. To reach their full potential, the demand for atomic-scale understanding of such interface-related phenomena has recently been increasing.

Among various materials, complex oxide heterostructures offer great opportunities to study interfacial phenomena because the strong coupling between the electronic, spin, and structural degrees of freedom in transition metal oxides leads to novel physical phenomena such as high-temperature superconductivity, colossal magnetoresistance, and multiferroicity [4]. In addition, numerous combinations of transition metal oxides can be realized with unit-cell-scale precision, as metaphorically compared with stacking LEGO blocks [5]. The most notable example is the LaAlO_3_/SrTiO_3_ (LAO/STO) heterostructure, in which the constituent materials are originally nonmagnetic band insulators. Since the discovery of metallic behavior with high mobility at the interface of LAO/STO [6], physical properties including superconductivity [7] and magnetism [8], which are unexpected from the bulk properties, have been reported. These results suggest that atomic-precision control of interface structures would open up a path for the exploration of new functional properties at surfaces and interfaces.

Here, we review our investigation of complex oxide surfaces using scanning tunneling microscopy/spectroscopy (STM/STS). The article is organized as follows. First, the STM system constructed for this research is briefly described. We then present STM/STS results on STO(001) substrate surfaces, for which the aim was to prepare atomically ordered surfaces. Subsequently, counterintuitive initial growth processes of STO, SrO, and LAO films on STO(001) are revealed. Next, we discuss atomically ordered spinel LiTi_2_O_4_ thin-film surfaces, which are extremely difficult to obtain in bulk single crystals and hence remain uncharted, to investigate their electronic and superconducting states. In the summary section, several challenges that remain to be overcome and future perspectives are reviewed.

## 2. STM integrated with pulsed laser deposition system

**Figure 1.**
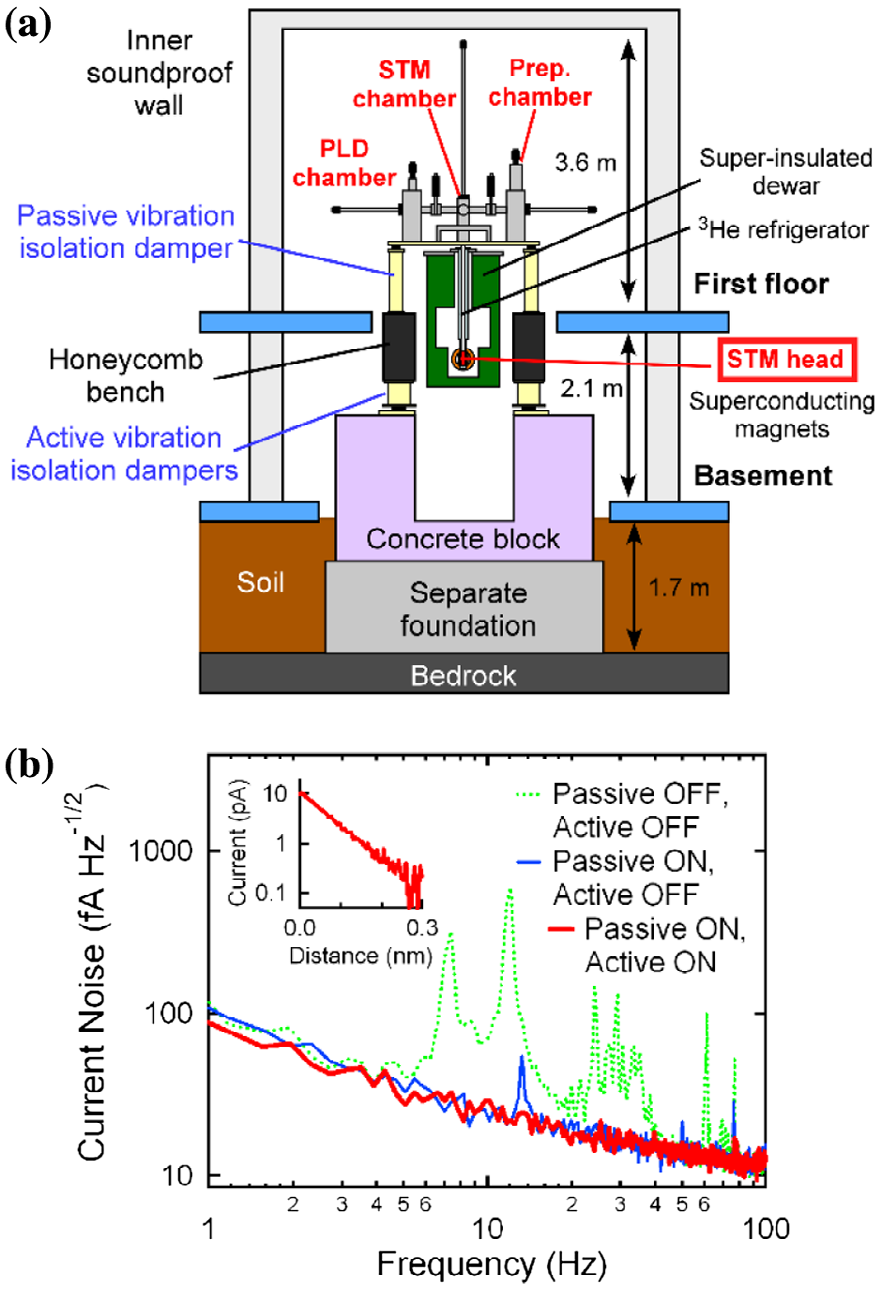
(a) Schematic diagram of the STM (scanning tunneling microscope) – PLD (pulsed laser deposition) system. (b) Vibration isolation effect on the tunneling current noise spectrum. The spectra were measured on a clean Si(001) surface at temperature *T* = 5 K with an open feedback loop. The feedback condition is a sample-bias-voltage *V*_s_ of +2 V and a tunneling current *I*_t_ of 10 pA.

Note: Adapted with permission from Iwaya et al., Rev. Sci. Instrum. 2011; 82: 083702. Copyright 2011 AIP Publishing.

To investigate atomic-scale surface structures and electronic states of oxide thin films, we first constructed a low-temperature STM integrated with a pulsed laser deposition (PLD) (STM–PLD system). The two apparatus are connected under ultrahigh vacuum (UHV) conditions to avoid surface contamination during sample preparation. We stress that the connection of the two systems is crucial because once the surface is contaminated, the intrinsic properties of the oxide surfaces are lost. The STM–PLD system consists of three UHV chambers: a PLD chamber, an STM tip preparation chamber, and a low-temperature STM chamber (Figure 1(a)). The STM system (Unisoku, USM-1300S, Osaka, Japan) is also equipped with a ^3^He refrigerator that can cool the STM down to temperature *T* = 0.4 K and with superconducting magnets that can apply magnetic fields up to 7 T vertically and 1 T along arbitrary directions (vector magnet). The PLD system is equipped with a reflection high-energy electron diffraction (RHEED) system for real-time monitoring of a deposition process and is evacuated by a turbomolecular pump and an ion pump. During STM measurements, the noisy turbomolecular pump is switched off while the ion pump is turned on to maintain UHV conditions.

We designed the STM–PLD system with two basic constraints. One is that the vibration noise levels during STM experiments must be as low as possible to obtain high-resolution data. The other is that outgassing from the components in the deposition chamber during thin-film growth must be suppressed as much as possible to obtain atomically ordered and chemically stable thin-film surfaces.

To satisfy the first requirement, we constructed a vibration isolation system on a foundation separated from the rest of the building. Active and passive vibration isolation dampers were installed on top of the separate foundation, and the STM was placed on the passive vibration isolation damper [9,10] (Figure 1(a)). In addition, the STM system was surrounded by double soundproof walls.

We designed the PLD system to be as compact as possible to suppress the transmittance of mechanical vibration to the STM; in other words, the resonant frequency of the system was designed to be as high as possible [11]. For instance, the target stage for PLD is rotated using piezo stacks instead of a typical stepping motor. Furthermore, an oxygen gas cylinder was placed directly on the passive vibration isolation table to reduce vibration transmission from outside of the vibration isolation table. These mechanical designs lead to an extremely stable tunneling condition where no characteristic vibration noises exist up to 100 Hz (Figure 1(b)).

The second requirement, the suppression of outgassing during thin-film growth, is critical to obtain atomically stable thin-film surfaces, although this point has almost been overlooked in the community in previous work. To satisfy this requirement, we heat the substrate by resistive heating (as applied for silicon) instead of using a typical radiative heater [12]. For insulating substrates, we found that metal films such as Pt deposited on the backside of the substrates work as heaters [12,13]. These resistive-heating methods significantly help to reduce outgassing from the surrounding when the sample is heated because the heated volume is limited to the substrate. More importantly, we place the substrate in an STM sample holder without using glue, which is well known to outgas at high temperatures and is likely to contaminate the sample surface. As a result, contamination of the surface from unwanted species is suppressed, and clean surfaces are obtained. Immediately after cooling the sample to room temperature, we transfer the samples to the STM head under UHV conditions.

## 3. SrTiO_3_ surfaces

First, we describe our results for the surface of STO(001) substrates. Single crystals of STO have been widely used as substrates for thin-film growth, ranging from perovskite oxides to even non-oxide materials such as iron-based superconductors and topological insulators. The advantage of using STO as a substrate is twofold. First, STO can either be a good insulator or metal; STO is known to exhibit a large dielectric constant suitable for an ideal gate insulator [14], whereas it can act as a metal with Nb doping suitable for a bottom electrode. Second, a unit-cell (UC)-scale abrupt step-and-terrace structure can be routinely achieved using well-established surface preparation processes [15]. It was thus reasonable to begin our research on STO substrate surfaces.

**Figure 2.**
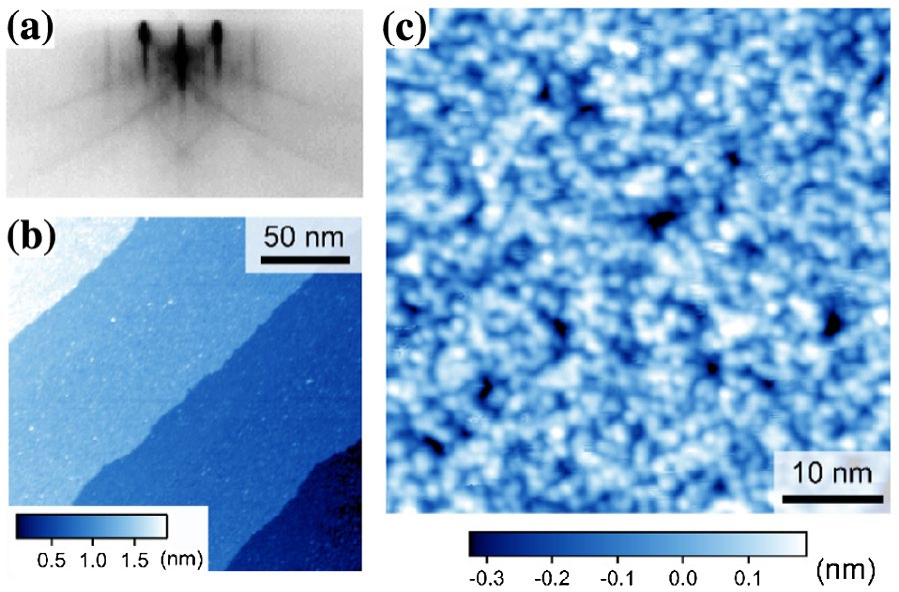
Conventional SrTiO_3_(001) surface. (a) Reflection high-energy electron diffraction (RHEED) pattern along the [110] azimuth. (b) Wide-scale STM image (200 × 200 nm^2^, *V*_s_ = +1.5 V, and *I*_t_ = 30 pA). (c) Magnified STM image (50 × 50 nm^2^, *V*_s_ = +2.8 V, and *I*_t_ = 30 pA). The STM images were obtained at 78 K.

Note: Adapted with permission from Ohsawa et al., J. Appl. Phys. 2010; 108: 073710. Copyright 2010 AIP Publishing.

A conventional STO(001) surface prepared using the standard annealing process surprisingly exhibited a disordered surface [16]. The typical RHEED pattern in Figure 2(a) exhibits (1 × 1) diffraction, and a wide-scale atomic force microscopy (AFM) image revealed clear step-and-terrace structures (not shown in the figure). These two results are conventionally cited in the literature as evidence of the ‘atomically ordered TiO_2_-terminated’ surface. Although we observed clear step-and-terrace structures using STM (Figure 2(b)), the atomic-scale surface structure was disordered (Figure 2(c)), raising the question of whether the widely accepted view of oxide thin-film growth based on an ideal surface termination is truly correct.

To obtain an atomically ordered STO surface, we investigated homoepitaxial STO films grown in two different modes, layer-by-layer and step-flow modes. In case that deposited atoms are strongly bound to the substrate rather than to each other, a monolayer tends to be formed before they develop into islands on the next layer; this growth mode is called ‘layer-by-layer mode’. By increasing growth temperature, atoms deposited on the surface diffuse to a step edge before they form islands. In this situation, the growing surface is seen as steps flowing across the surface, and hence the growth mode is called ‘step-flow mode’ [17].

**Figure 3.**
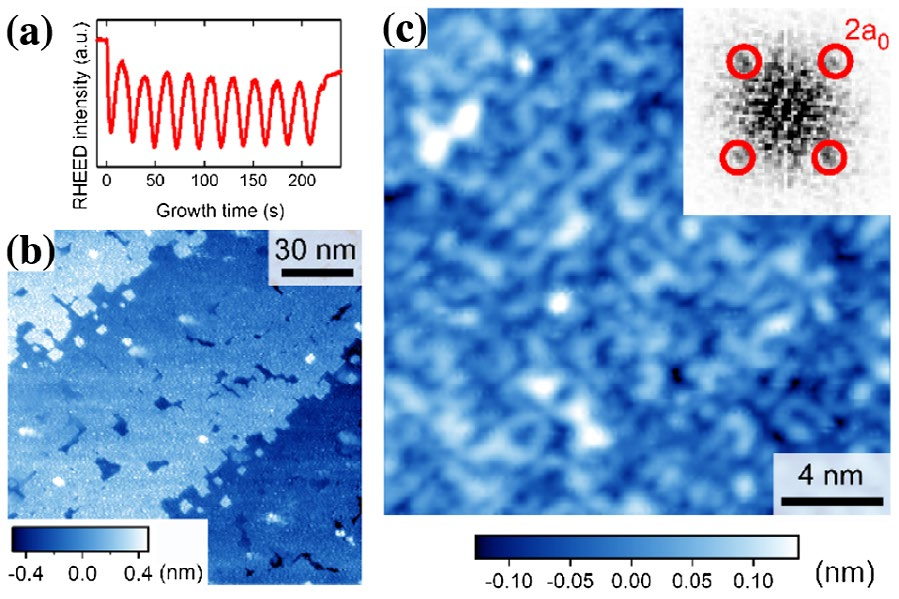
SrTiO_3_(001) film grown in the layer-by-layer mode. (a) RHEED oscillation for a 10-UC (unit cell)-thick SrTiO_3_ film. (b) Wide-scale STM image of a 10-UC-thick SrTiO_3_ film (150 × 150 nm^2^). (c) Magnified STM image (20 × 20 nm^2^, *V*_s_ = +2.0 V, and *I*_t_ = 20 pA). Inset: fast Fourier transform (FFT) image showing the (2 × 2) reconstructed structure. The STM images were obtained at 78 K.

Note: Adapted with permission from Ohsawa et al., J. Appl. Phys. 2010; 108: 073710. Copyright 2010 AIP Publishing.

The layer-by-layer growth was readily identified by sinusoidal RHEED intensity oscillations during film depositions (Figure 3(a)), which enabled us to count the number of UCs deposited on the substrate. The wide-scale STM image of 10-UC-thick STO(001) in Figure 3(b) revealed a meandering step structure with a height of 1 UC (0.4 nm) and a terrace with pits and islands of a few nanometers in size. Focusing on a smaller scale, we observed that a (2 × 2) surface reconstruction was formed along with a number of defects (Figure 3(c)). Because a similar surface structure was also observed in a 50-UC-thick film, the (2 × 2) reconstruction is possibly the most stable surface structure of the layer-by-layer grown STO(001) films under our growth conditions.

**Figure 4.**
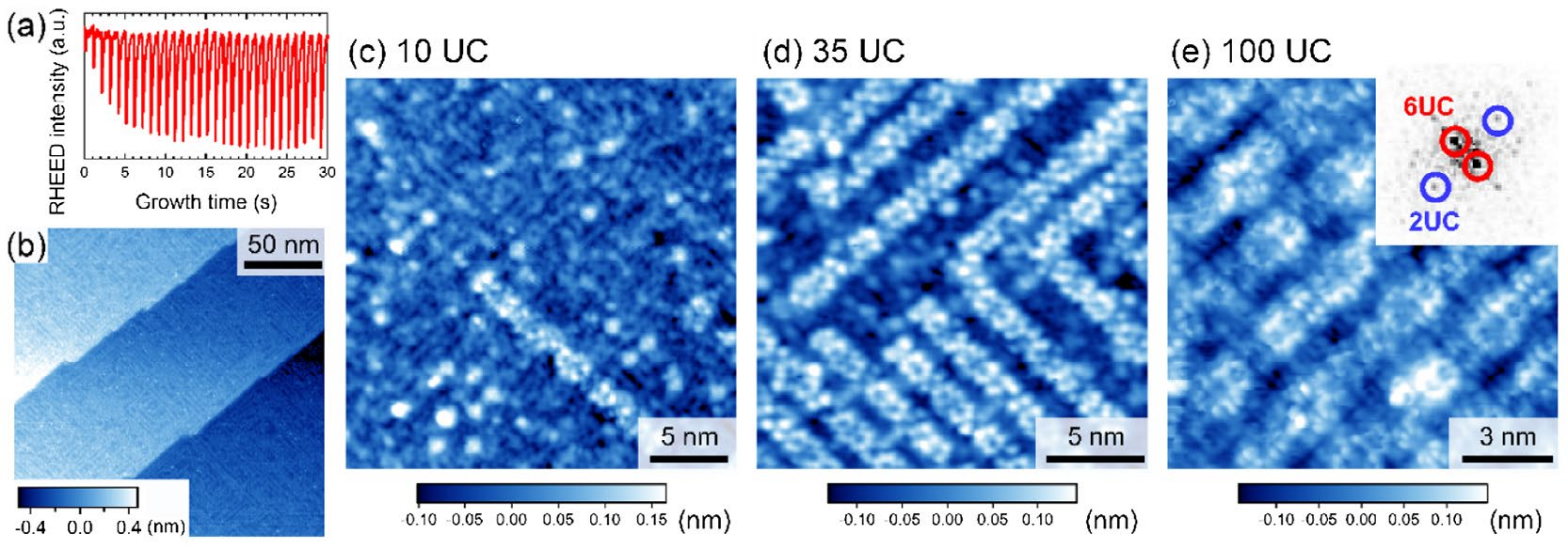
SrTiO_3_(001) film grown in the step-flow mode. (a) Typical RHEED oscillations during the step-flow growth mode. (b) Wide-scale STM image of 35-UC-thick SrTiO_3_ film (200 × 200 nm^2^, *V*_s_ = +2.0 V, and *I*_t_ = 40 pA). (c)–(e) Thickness dependence of the surface structure: (c) 10-UC thick (25 × 25 nm^2^), (d) 35-UC thick (20 × 20 nm^2^), and (e) 100-UC thick (13 × 13 nm^2^). A FFT image of (e) showing the (6 × 2) reconstructed structure is displayed in the inset. All the STM images were obtained at 78 K.

Note: Adapted with permission from Iwaya et al., Appl. Phys. Express. 2010; 3: 075701. Copyright 2010 The Japan Society of Applied Physics.

**Figure 5.**
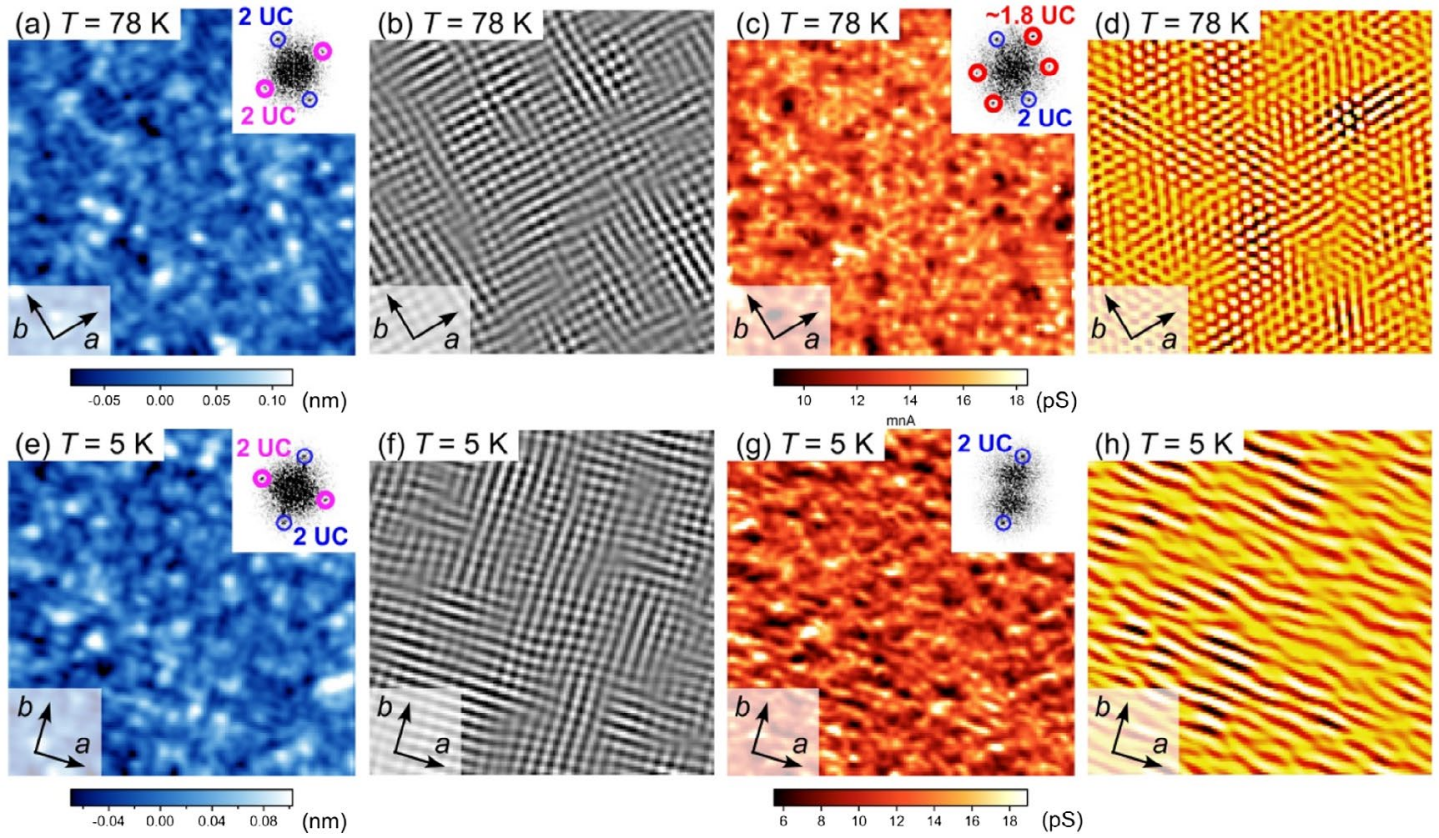
Electronic liquid states at the SrTiO_3_ thin-film surface. (a) STM image of a SrTiO_3_(001) film grown in the step flow mode (*T* = 78 K, *V*_s_ = +1.5 V, *I*_t_ = 30 pA). A FFT image showing the (2 × 1)−(1 × 2) domain structure is presented in the inset. (b) Fourier-filtered STM image created using the (2 × 1)−(1 × 2) peaks in (a). (c) d*I*_t_ */*d*V*_s_ map at *V*_s_ = +1.0 V obtained simultaneously with (a). A FFT image indicating the hexagonal-like electronic modulation is displayed in the inset. (d) Fourier-filtered STM image of (c) created using the hexagonal-like peaks. (e) STM image of a STO(001) film measured at *T* = 5 K, *V*_s_ = +1.5 V, and *I*_t_ = 30 pA. The film was grown under the same conditions as that in (a). A FFT image is shown in the inset. (f) Fourier-filtered STM image created using the (2 × 1)−(1 × 2) peaks in (e). (g) d*I*_t_ */*d*V*_s_ map at *V*_s_ = +1.0 V obtained simultaneously with (e). A FFT image indicating the uniaxial electronic modulation is shown in the inset. (h) Fourier-filtered image of (g) using the uniaxial peaks. All the images span 20 × 20 nm^2^.

We next examined homoepitaxial STO thin films grown in the step-flow mode [18]. In contrast to the layer-by-layer mode, the RHEED intensity exhibited an oscillatory behavior characterized by an exponential recovery to the initial intensity level (Figure 4(a)). The wide-scale STM image of a 35-UC-thick film in Figure 4(b) reveals a clear step-and-terrace structure with a single UC height, which is characteristic of thin-film surfaces grown in the step-flow mode. On a smaller scale, we observed that one-dimensional (6 × 2) nanostructures were preferentially grown along the *a* or *b* axis and that the density of the nanostructures increased with increasing film thickness (Figure 4(c)–(e)). Considering the similar nanostructure reported for a single-crystal STO(001) surface prepared by sputtering and annealing [19], we attributed the observed nanostructure to a TiO_*x*_-based structure. We also observed that the one-dimensional nanostructures were metastable because they disappeared after post-deposition annealing at ~1400 K. The surface after the post-deposition annealing exhibited a complicated domain structure with (2 × 1) and (1 × 2) reconstructions (Figure 5(a)) identified as fourfold symmetry in a fast Fourier transform (FFT) of the STM image (Figure 5(a), inset).

To more clearly visualize this domain structure, we present an inverse FFT-filtered image in Figure 5(b) created by selecting the (2 × 1) and (1 × 2) peaks in the FFT image. We observed intriguing electronic modulations on this surface. Figure 5(c) presents a d*I*_t_/d*V*_s_ conductance map obtained from the same field of view as the STM image in Figure 5(a) (*T* = 78 K), where *I*_t_ is the tunneling current and *V*_s_ is the bias voltage applied to the sample. The symmetry difference between the surface structure (STM image) and its electronic state (conductance map) is striking compared with the respective FFT images (Figure 5(a) and (c), insets). Although the peaks corresponding to the (1 × 2) reconstruction are observed in both the STM image and conductance map, the peaks corresponding to the (2 × 1) reconstruction are hardly visible in the conductance map. Instead, new peaks with a period of ~1.8 UC (= 0.7 nm) and a rotation angle of ~60° are observed only in the conductance map, yielding an overall *C*_2v_ symmetry. The symmetry difference in real space between the surface structure and conductance map can be appreciated by comparing the corresponding inverse FFT-filtered images in Figure 5(b) and (d).

We also investigated the surface at lower temperature. Figure 5(e) presents an STM image at *T* = 5 K of the film grown under the same conditions as the film shown in Figure 5(a). Although the surface retained the same (2 × 1)−(1 × 2) structure and symmetry (Figure 5(e), inset), its conductance map shows another striking symmetry change (Figure 5(g)). As observed in the FFT image (Figure 5(g), inset), the conductance map exhibited uniaxial modulation along the *a* or *b* axis (*C*_2v_ symmetry), which is different from the surface structure and from the observations at *T* = 78 K. The temperature dependence of the modulation is evident upon comparing the two inverse FFT-filtered images (Figure 5(d) and (h)). Since the symmetry change is clearly identified only in the conductance maps, the origin of this phenomenon is electronic rather than a structural evolution. The (2 × 1)−(1 × 2) reconstructed surface was also examined using transmission electron microscopy (TEM), and a 2D TiO_*x*_-rich structure, completely different from the bulk structure, was proposed [20]. Based on these results, we suggest that a 2D electronic state is formed near the surface where enhanced electron correlation is manifested as the electronic liquid-crystal-like modulations. To discuss the origin in details, temperature dependence of the modulation needs to be carefully investigated in the future. These results demonstrate the possibility that intriguing electronic phenomena could be induced by tailoring atomic-scale structures at transition-metal-oxide surfaces [21].

**Figure 6.**
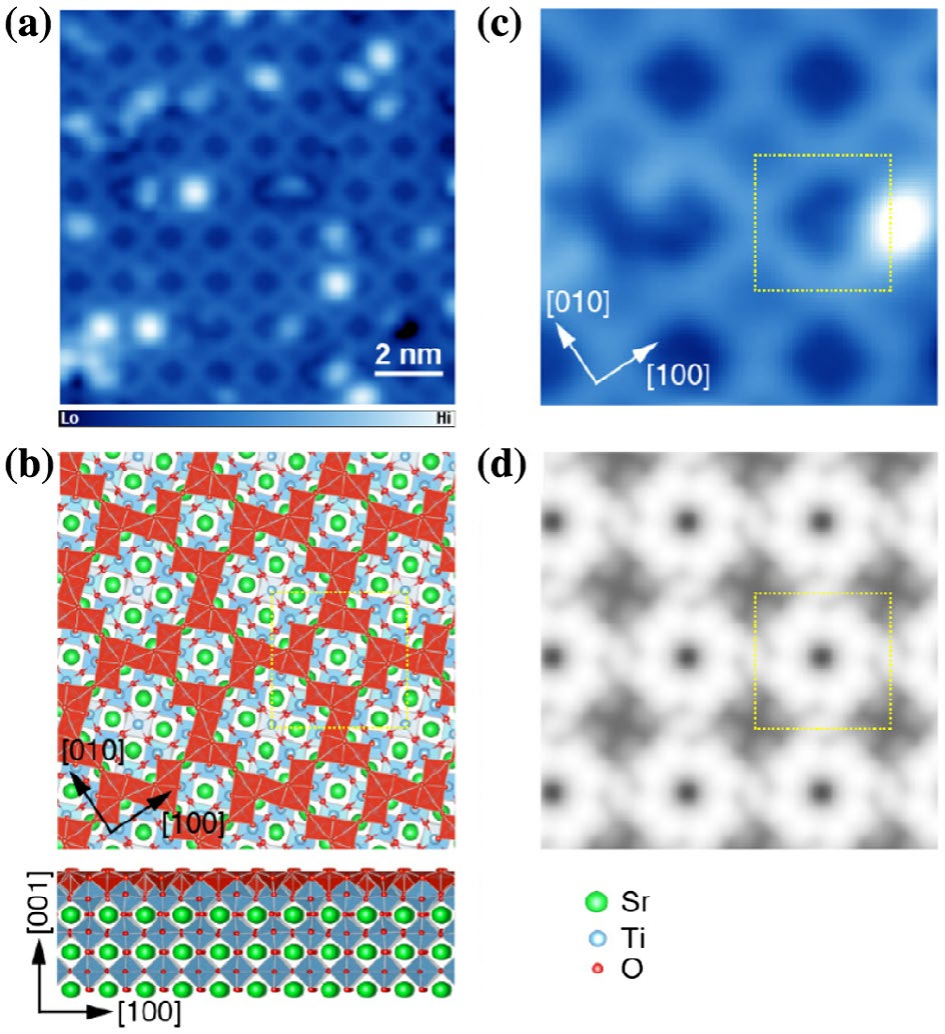
SrTiO_3_(001)−(√13 × √13)-*R*33.7° reconstructed surface. (a) STM image (11.5 × 11.5 nm^2^, *V*_s_ = +1.5 V, *I*_t_ = 30 pA, *T* = 5 K). (b) Magnified image (3.7 × 3.7 nm^2^). (c) The surface structure of the reconstructed surface. TiO_6_ octahedra in bulk and truncated octahedra TiO_5_ (TiO_2_ nanomesh) at the surface are shown in blue and red, respectively. (d) Simulated STM image at *V*_s_ = +1.5 V. The dotted square encloses a (√13 × √13)-*R*33.7^o^ surface unit cell.

Note: Reprinted with permission from Hamada et al., J. Am. Chem. Soc. 2014; 136: 17201–17206. Copyright 2014 American Chemical Society.

We also attempted to obtain an atomically ordered STO surface by improving the well-established surface preparation processes based on chemical etching and annealing. By suppressing oxygen deficiencies in a bulk crystal by annealing, a STO(001) (√13 × √13)−*R*33.7° (hereafter called (√13 × √13) for brevity) reconstructed surface (Figure 6(a)) was reproducibly obtained and remained stable during subsequent thin-film deposition [12,22]. We also determined the atomic arrangement of the (√13 × √13) reconstructed surface by combining STM and density functional theory calculations [23]. In our model shown in Figure 6(b), the Z-shaped framework is composed of five edge-sharing truncated octahedra (TiO_5_) units, which are connected via the corner of TiO_5_, forming a two-dimensional network on the TiO_2_-terminated surface (called TiO_2_ nanomesh hereafter). This can be considered as a TiO_2_ adlayer with small and large vacant sites. The model developed in this work coincides with that proposed based on TEM experiments [24]. Using this model, the STM image was successfully reproduced using first-principles calculations (Figure 6(c) and (d)), and accordingly, the STM image was understood in terms of nondegenerate Ti 3d orbital states originating from broken inversion symmetry at the surface.

## 4. Initial growth processes of STO, SrO, and LAO films on STO(001)

The initial growth processes of transition-metal-oxide thin films strongly affect the formation of interface structures. Despite their importance, these processes have only been characterized using diffraction methods such as RHEED measurements; hence, the atomic-scale view of the initial stage of thin-film growth has remained unclear. We atomically resolved the initial growth processes of STO, SrO, and LAO films on STO(001) substrates using STM. The (√13 × √13)-reconstructed STO surface described in the previous section was used as a substrate for all of the thin-film growth because of its robustness under a wide range of growth conditions [25,26].

**Figure 7.**
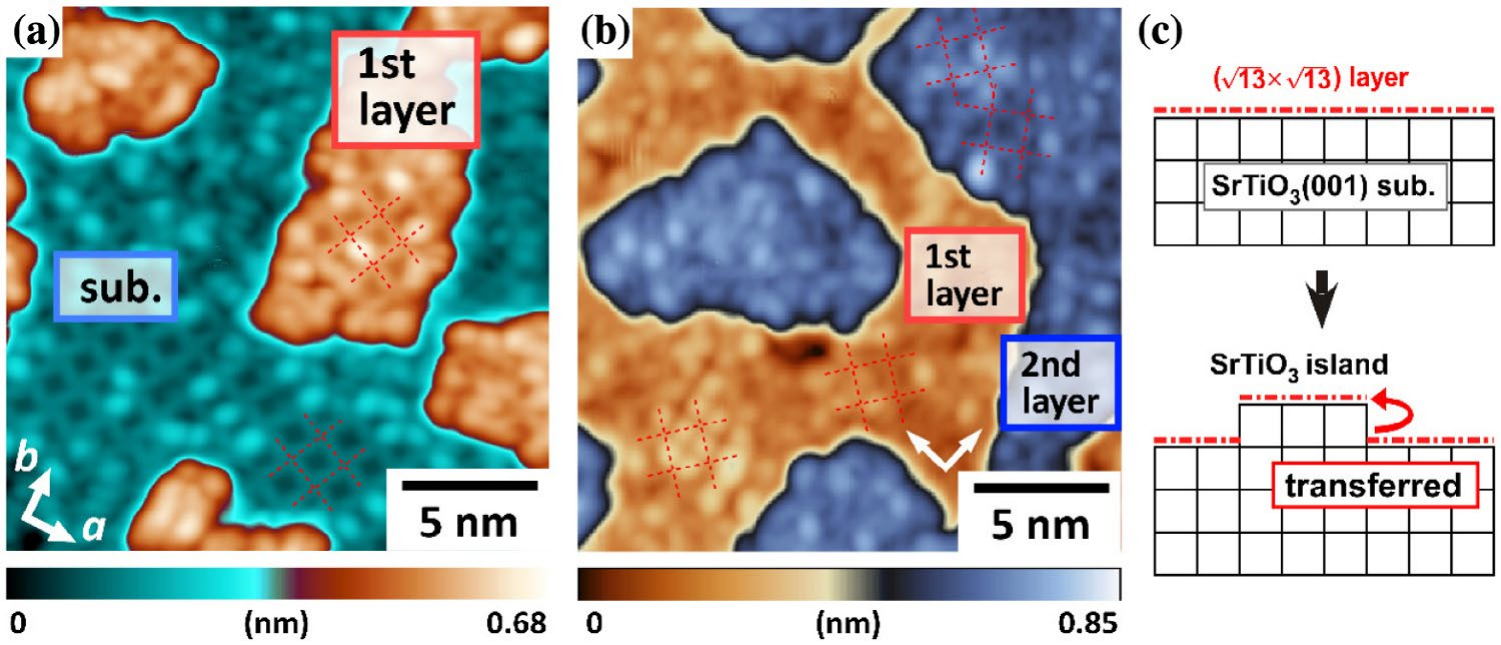
STM images of ultrathin SrTiO_3_(001) films grown on (√13 × √13)-*R*33.7^o^ reconstructed surfaces. Thickness of (a) 0.3 UCs and (b) 1.6 UCs. The images were obtained at 78 K (20 × 20 nm^2^, *V*_s_ = +1.5 V, *I*_t_ = 30 pA). (c) Schematic illustration of the homoepitaxial growth mechanism of SrTiO_3_ film on the (√13 × √13)-*R*33.7° substrate.

Note: Reprinted with permission from Shimizu et al., ACS Nano 2011; 5: 7967–7971. Copyright 2011 American Chemical Society.

As a prototypical example, homoepitaxial STO thin films grown on a STO(001) substrate were investigated. We observed the (√13 × √13) structures all over the surfaces of 1-UC-thick and 2-UC-thick STO islands (Figure 7(a) and (b)). In analogy with the homoepitaxial growth of GaAs thin films [27], we suggest that the atomic structure of the substrate surface, the TiO_2_ nanomesh, is spontaneously transferred to the STO film surface, implying atomic-scale coherent epitaxy at the interface between the STO thin film and substrate (Figure 7(c)).

**Figure 8.**
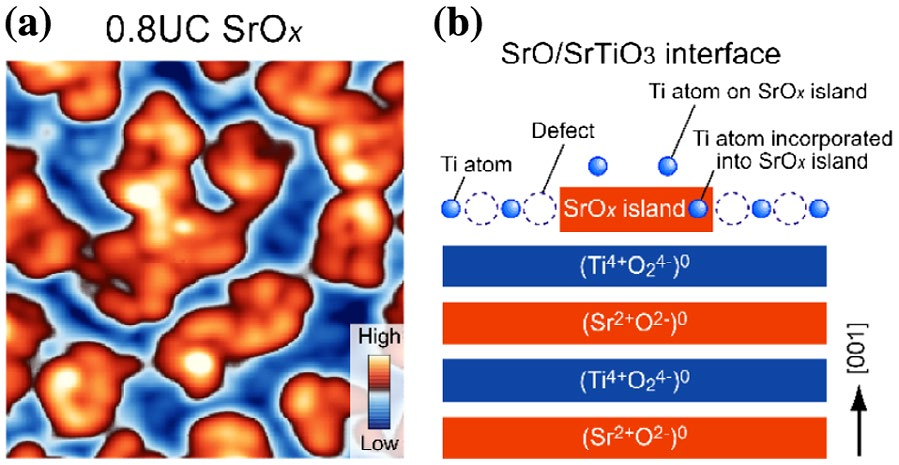
SrO_*x*_ film grown on SrTiO_3_ (001)−(√13 × √13)-*R*33.7° reconstructed substrate surface. (a) STM image of 0.8-UC-thick SrO_*x*_ film (15 × 15 nm^2^, *V*_s_ = +1.9 V, *I*_t_ = 30 pA, *T* = 78 K). (b) Schematic illustration of the SrO_*x*_-deposited SrTiO_3_(001)−(√13 × √13)-*R*33.7° reconstructed surface. The formation of SrO_*x*_ islands induces the generation of defects on the surrounding substrate surface. Excess Ti ions on the (√13 × √13)-*R*33.7^o^ surface are distributed on top of the SrO_*x*_ island or incorporated into the SrO_*x*_ island.

Note: Adapted with permission from Ohsawa et al., ACS Nano 2014; 8: 2223–2229. Copyright 2014 American Chemical Society.

We next investigated SrO films grown on STO(001). The growth of SrO is motivated by the fact that the metallic conductivity at the LAO/STO interface is distinctly suppressed when the heterostructure is fabricated on a SrO-terminated STO substrate [28]. Because the origin of this suppression is not fully understood, it is critical to understand the growth processes and atomic structures of a SrO-terminated surface. We observed that sub-UC-thick SrO_*x*_ films were not characterized by atomically ordered surface structures [29] (Figure 8(a)) in marked contrast to homoepitaxial STO films. In addition, some parts of the SrO_*x*_ islands exhibited striking bias-dependent height changes in STM images (relative to the substrate surface) [30], whereas the rest of the island exhibited almost no bias dependence. This result indicates that the composition of SrO_*x*_ islands is not spatially uniform and that a certain amount of Ti atoms is possibly randomly incorporated into the SrO_*x*_ islands. Moreover, the formation of SrO_*x*_ islands was observed to introduce defects into the surrounding STO substrate surface. Based on these results, we suggest possible atomic-scale SrO_*x*_ island formation on the (√13 × √13) substrate surface in Figure 8(b). This contrasting growth behavior between SrO and STO films may provide a clue toward elucidating the origin of the interface-dependent transport properties of the LAO/STO interface.

**Figure 9.**
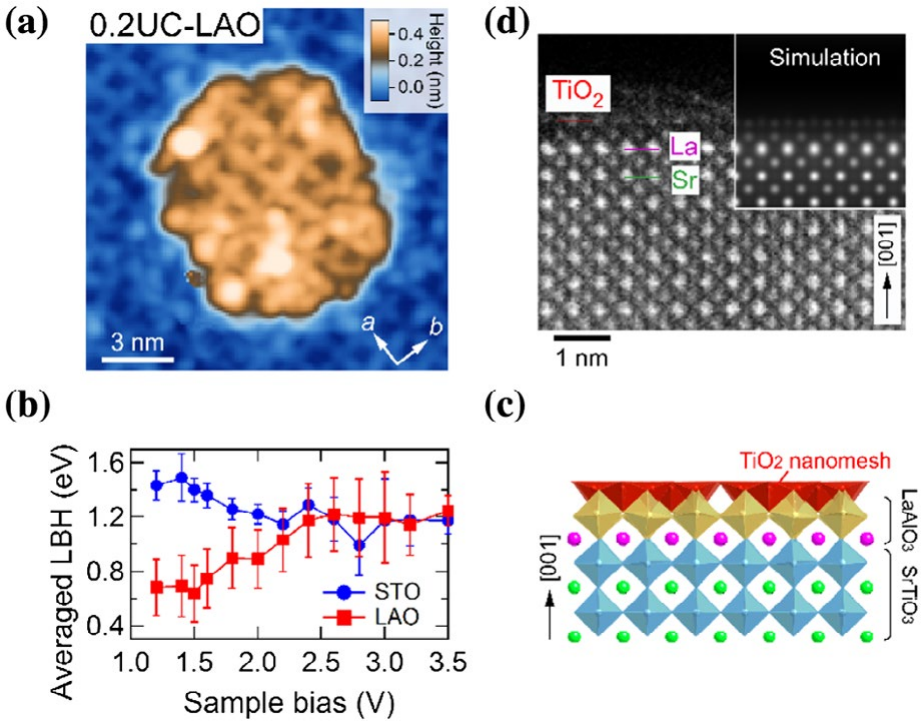
LAO film on STO(001)-(√13 × √13)-*R*33.7° substrate surface. (a) STM image of 0.2-UC-thick LAO film (15 × 15 nm^2^, *V*_s_ = +1.2 V, *I*_t_ = 30 pA, *T* = 78 K). (b) Averaged LBH of the LAO island and STO substrate surface as a function *V*_s_. (c) Model of the LAO/STO interface (TiO_2_ nanomesh/AlO_2_/LaO/bulk TiO_2_). (d) High-angle annular dark-field scanning TEM image of 1-UC-thick LAO film on the STO(001)-(√13 × √13)-*R*33.7° substrate. A simulation image is shown in the inset.

Note: Adapted with permission from Ohsawa et al., ACS Nano 2015; 9: 8766–8772. Copyright 2015 American Chemical Society.

Regarding the LAO/STO interface, the initial growth processes of LAO thin films on STO(001) were investigated using STM [31]. Interestingly, the STM image of the LAO islands revealed a (√13 × √13) structure (Figure 9(a)), as observed in the STO film on the STO substrate (Figure 7). In addition, no significant differences were observed between the d*I*_t_ */*d*V*_s_ spectra measured at the LAO island and those at the STO substrate. These results indicate that the LAO island cannot be distinguished from the STO island in terms of STM images and d*I*_t_ */*d*V*_s_ spectra.

To detect any signatures derived from LAO, we performed local barrier height (LBH) measurements, which are sensitive to the atomic species on the surface. The averaged LBH on the LAO islands showed a different bias dependence from that on the STO substrate surface (Figure 9(b)). The LBH of the (√13 × √13) STO surface gradually decreased with increasing *V*_s_, which is typical behavior resulting from the biasing effect of the tunneling barrier, as previously observed on conventional semiconductor surfaces [32]. In contrast, the LBH of the LAO island exhibited an opposite bias dependence, increasing with *V*_s_ and eventually exhibiting almost the same values as those on the (√13 × √13) STO surface in the *V*_s_ range of 2.5–3.5 V (Figure 9(b)). Considering that a tunneling current at high *V*_s_ is generally sensitive to the surface rather than the subsurface because the distance between the STM tip and surface is larger at higher *V*_s_, we suggest that the surface structure of the LAO island is identical to that of the (√13 × √13) substrate surface, that is, a TiO_2_ nanomesh. The results suggest that the LAO monolayer is deposited on the TiO_2_-terminated STO surface, preserving AO/BO_2_ stacking of the ABO_3_ perovskite (Figure 9(c)). This scenario was indeed supported by our scanning TEM measurements (Figure 9(d)). The unusual bias dependence of the LBH of the LAO island possibly results from a negatively charged AlO_2_ layer under the TiO_2_ nanomesh.

The single-atom-thick TiO_2_ nanomesh is analogous to graphene, and we expect unique properties to arise from such oxide nanostructures. We recently discovered that such an atomically controlled LAO/STO interface significantly affects transport properties compared with the conventional LAO/STO interface such as reduced critical thickness for the metallic interface and enhanced magnetoresistance [33]. We note that the TiO_2_ nanomesh cannot be fabricated by the deposition of TiO_2_ on LAO, as it is widely known that anatase TiO_2_ is formed on LAO surfaces [34,35]. Therefore, atomic-scale understanding of growth processes is of great importance.

## 5. Atomic structures and anomalous superconductivity on LiTi_2_O_4_ surfaces

Motivated by recent discoveries of unusual superconductivity at LAO/STO interfaces [36] and in ultrathin FeSe films [37], we investigated the superconducting state of spinel oxide LiTi_2_O_4_ (LTO) thin-film surfaces [38]. LTO exhibits the highest superconducting transition temperature *T*_c_ of 13.7 K among spinel superconductors and possesses a large degeneracy of charge, spin, and orbital states in bulk form [39,40]. Because we expect a prominent degeneracy lifting of such electron degrees of freedom at the surface, LTO offers an intriguing platform to explore emergent surface superconductivity.

**Figure 10.**
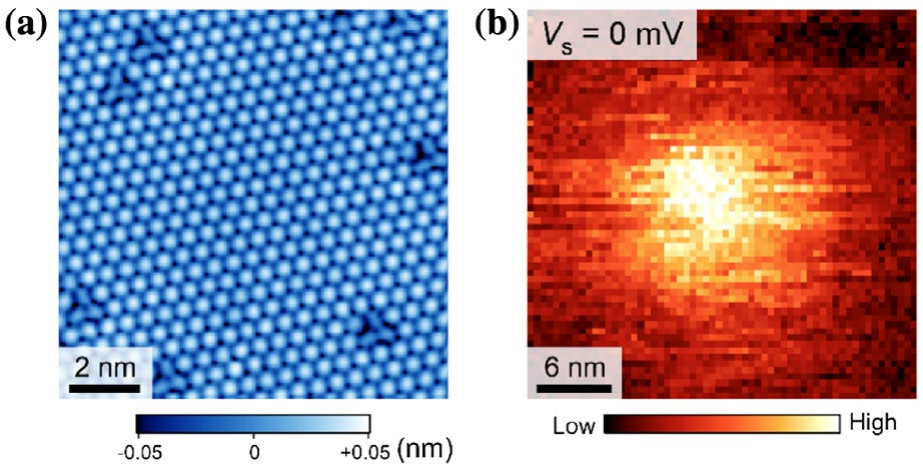
Atomically resolved LiTi_2_O_4_(111) thin-film surfaces. (a) STM image (11 × 11 nm^2^, *V*_s_ = +30 mV, *I*_t_ = 30 pA, *T* = 4.2 K). (b) d*I/*d*V* map at a single vortex core (31.5 × 31.5 nm^2^). The results were obtained at *T* = 4.2 K with a magnetic field of 1.5 T. Set point: *V*_s_ = −10 mV, *I*_t_ = 30 pA.

Note: Adapted with permission from Okada et al., Nat. Commun. 2017; 8: 15975. Copyright 2017 Nature Publishing Group.

A well-ordered triangular lattice, as expected from the unit cell of LTO(111), was clearly observed, indicating no significant surface reconstruction (Figure 10(a)). We found that 2*Δ*/*k*_B_ *T*_c_ estimated at the surface was approximately 3.0 (assuming a bulk *T*_c_ of 13 K), which is substantially smaller than the bulk value (2*Δ*/*k*_B_ *T*_c_ = 3.5–4.0) [41]. Here, *Δ* and *k*_B_ represents the superconducting gap amplitude and the Boltzmann constant, respectively. Correspondingly, the coherence length (*ξ* = 12.4 nm) estimated from the spatial distribution of the vortex core state shown in Figure 10(b) was remarkably larger than that obtained from the upper critical field *H*_c2_ (*ξ* = 4.1–4.7 nm). These results indicate suppressed superconductivity at the surface. Our first-principles calculations revealed that the surface density of states at the Fermi energy was smaller than that of the bulk due to a pseudogap opening originating from unique surface termination consisting of TiLi_2_ layer [38]. The reduction in the density of states possibly causes the suppressed surface superconductivity, as naively expected from the Bardeen–Cooper–Schrieffer theory. To discuss the relations between such modified superconductivity and frustration effects on the surface in further detail, superconducting states at lower temperatures need to be investigated in the future.

## 6. Conclusions and outlook

In this review, we discussed STM studies of several transition-metal oxide thin films, with the aim of revealing the atomic-scale structures and electronic states of those surfaces. Although we have paved the way to study oxide surfaces with atomic resolution, many unresolved issues remain to be overcome, as discussed below.

### 6.1. Crystal orientation dependence of atomic and electronic structures

Considering that the electronic states of metal oxide systems are generally anisotropic because of the directionality of *p* and *d* orbitals, we expect significant orientation-dependent physical properties. Thin-film growth techniques allow us to prepare surfaces with different crystal orientations. In this review, we mainly focused on the (001) plane in the cubic perovskite structures and the (111) plane in the spinel structure; however, other orientations such as the (111) and (110) planes in cubic perovskite structures also offer interesting platforms to explore surface/interface-related phenomena. In addition, layered metal oxides possessing the K_2_NiF_4_ structure are attractive because the cross-section of CuO_2_ planes in high-temperature cuprate superconductors, such as La_2−*x*_Sr_*x*_CuO_4_, or of CoO_2_ planes in ferromagnetic metal Sr_2_CoO_4_ [42] can be investigated using (100)-oriented LaSrAlO_4_ substrates.

### 6.2. Properties of one- and two-dimensional oxide nanostructures

Nanostructures such as one-dimensional nanoribbons are known to grow on oxide surfaces [43,44]; however, their atomic structures and electronic states have remained almost unexplored. Furthermore, we have shown that two-dimensional structures could be formed on oxide surfaces [31]. By utilizing atomically ordered oxide surfaces as discussed in this review, it would be possible to fabricate atomically ordered nanostructures, allowing us, for instance, to investigate electronic and magnetic properties in low-dimensional structures or to realize spatially modulated superconducting states. To determine the atomic arrangements of nanostructures, it is indispensable to combine STM experiments with first-principles calculations, although the size of the supercell may be too large to handle even for currently available high-performance computing systems. To overcome this difficulty, further theoretical developments including an increase of computational power are highly demanded.

### 6.3. Surface chemistry

There remain many unsettled issues involving surface chemistry. For instance, catalysts, batteries, and fuel cells are not yet fully understood at the microscopic scale. (La,Sr)MnO_3_ and (La,Ca)MnO_3_ are electrode materials for fuel cells; thus, the atomically ordered surface should be ideal to study the atomistic dissociation processes of oxygen molecules on the surface. Furthermore, various metal oxides have been practically used as catalyst materials; however, atomic-scale investigations of the catalytic reaction have remained challenging. More fundamentally, the interaction between molecules and an oxide thin-film surface including defects and nanostructures should be understood at the atomic scale.

To address these issues, the first important step is to find suitable materials that exhibit atomically ordered surfaces. In fact, we cannot expect to obtain atomic-resolution STM images of all oxide materials; however, we believe that many oxide materials remain that are likely to exhibit atomically ordered surfaces. Empirically, oxide thin-film surfaces whose electronic band dispersions are clearly observed by angle-resolved photoemission spectroscopy are promising for STM. Furthermore, in our experience, thin films exhibiting spiral surface structures or extremely large terraces even with step bunching in AFM images are worthy of investigation.

Finally, we note that because of recent improvements in pumping systems, it is relatively easy to build a portable UHV suitcase chamber with a typical base pressure of ~1 × 10^−10^ Torr using a combination of an ion pump and getter pump. Such suitcase chambers will allow us not only to conduct efficient experiments but also to extend STM capabilities to a wider range of thin films in the near future. The combination of multiple characterization methods for the same oxide samples would certainly contribute to a better understanding of oxide surfaces.

## Disclosure statement

No potential conflict of interest was reported by the authors.

## Funding

This work was supported by the World Premier Research Institute Initiative promoted by the Ministry of Education, Culture, Sports, Science and Technology (MEXT) of Japan. T. H. acknowledges funding from JSPS KAKENHI [grant numbers 26246022; 26106502; 26108702 and 26610092] from the JSTPRESTO and JST-CREST programs.
